# From dilute isovalent substitution to alloying in CdSeTe nanoplatelets†
†Electronic supplementary information (ESI) available: TEM, XRD, TA and PLE experimental results. Detailed analysis of the PL fit of alloyed NPLs and spectral peak asymmetry of the doped NPLs. See DOI: 10.1039/c6cp01177b



**DOI:** 10.1039/c6cp01177b

**Published:** 2016-05-05

**Authors:** Ron Tenne, Silvia Pedetti, Miri Kazes, Sandrine Ithurria, Lothar Houben, Brice Nadal, Dan Oron, Benoit Dubertret

**Affiliations:** a Department of Physics of Complex Systems , Weizmann Institute of Science , 76100 Rehovot , Israel . Email: miri.kazes@weizmann.ac.il; b ESPCI ParisTech , PSL Research University , CNRS , Sorbonne Universités , UPMC Univ. Paris 6; LPEM , 10 rue Vauquelin , F-75231 Paris Cedex 5 , France; c Nexdot , 10 Rue Vauquelin , 75005 Paris , France; d Department of Chemical Research Support , Weizmann Institute of Science , 76100 Rehovot , Israel

## Abstract

Cadmium chalcogenide nanoplatelet (NPL) synthesis has recently witnessed a significant advance in the production of more elaborate structures such as core/shell and core/crown NPLs. However, controlled doping in these structures has proved difficult because of the restrictive synthetic conditions required for 2D anisotropic growth. Here, we explore the incorporation of tellurium (Te) within CdSe NPLs with Te concentrations ranging from doping to alloying. For Te concentrations higher than ∼30%, the CdSe_*x*_Te_(1–*x*)_ NPLs show emission properties characteristic of an alloyed material with a bowing of the band gap for increased concentrations of Te. This behavior is in line with observations in bulk samples and can be put in the context of the transition from a pure material to an alloy. In the dilute doping regime, CdSe:Te NPLs, in comparison to CdSe NPLs, show a distinct photoluminescence (PL) red shift and prolonged emission lifetimes (LTs) associated with Te hole traps which are much deeper than in bulk samples. Furthermore, single particle spectroscopy reveals dramatic modifications in PL properties. In particular, doped NPLs exhibit photon antibunching and emission dynamics significantly modified compared to undoped or alloyed NPLs.

## Abstract 2

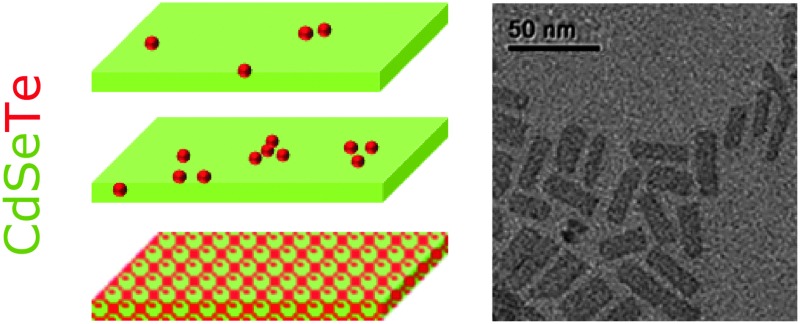
Synthesis and spectroscopy of CdSe_*x*_Te_(1–*x*)_ nanoplatelets going from the alloyed regime to dilute doping.

## Introduction

Colloidal quantum wells (QWs), also termed nanoplatelets (NPLs), are a new class of quantum confined crystal architectures that has emerged in recent years as part of the prolific development in colloidal synthesis of semiconductor (SC) nanocrystals. NPLs have a well-defined thickness of only a few atomic monolayers and lateral dimensions of tens of nanometers with defect-free crystallinity.^1^ While some of the optical and electronic properties of NPLs are reminiscent of those of 1D quantum confined systems (*i.e.* a quantum well), others, particularly those involving multicarrier interactions can be more characteristic of 3D confined systems (*i.e.* quantum dots) due to the inherently high carrier density upon optical excitation.^2^

The characteristic absorption of cadmium chalcogenide SC NPLs shows two sharp excitonic transitions from the heavy hole (HH) and the light hole (LH) valance bands to the conduction band.^3^ The large surface to volume ratio and the smaller dielectric constant of the media surrounding the NPL result in a reduced excitonic radius and a large binding energy (∼100 s of meV).^4^ This, in turn, gives rise to a short radiative lifetime and a small Stokes shift. Shorter radiative lifetimes can help better compete with Auger recombination whose rate is slowed down due to the continuous density of states. As a result, the probability for multiple photon emission by a single particle is increased, leading to the use of NPLs in optical gain devices.^5,6^

While these structures have unique optoelectronic properties, the variety of NPLs is still considerably small compared with their 3D counterparts limiting the tunability of their properties. To date, CdS, CdSe and CdTe NPLs as well as heterostructures in both core/shell and core/crown geometries have been synthesized.^7,8^ Sandwich-like core/shell structures have been shown to exhibit not only luminescence spectral tunability, but also enhanced quantum efficiency and better stability.^9,10^ Type-II core/crown heterostructure NPLs such as CdSe/CdTe and CdTe/CdSe have enabled us to extend NPL emission to the near-infrared spectral region.^11–14^ Yet, in both core/shell and core/crown NPLs, a few of the unique properties such as the short radiative lifetime and the narrow luminescence spectrum of NPLs are somewhat compromised.^7,15^ This results either from the breaking of translational symmetry or the reduction of quantum confinement. Since few-layer thick NPLs of a given material only offer discrete emission bands, a new mechanism for tuning the NPL emission while maintaining both strong confinement and translational symmetry is desired. One pathway towards achieving this objective is *via* isovalent substitution or alloying as in the CdSe_*x*_Te_(1–*x*)_ system, whose bulk band gap can be tuned from 1.4 eV to above 1.7 eV by composition tuning (going from *x* ∼ 0.5 to *x* = 1) due to band gap bowing.^16,17^ CdSeTe alloyed QDs have been successfully fabricated^18–20^ and recently utilized as sensitizers in record QD sensitized solar cells exhibiting an overall efficiency of over 8%.^21,22^ Working in the dilute Te-doping regime, it was shown, both experimentally and theoretically, that for strongly confined structures the electrons remain delocalized across the QDs, while the holes are strongly localized around the Te atoms, significantly modifying the spectroscopic behavior.^23^ In particular, doping results in large exciton–exciton repulsion, thus increasing the rate of Auger recombination. Another result of an increased interaction term is the elimination of the biexciton–exciton degeneracy, making the QDs an effective three-level system which is desirable for many optical applications such as optical gain.^24,25^ It is therefore natural to expect the CdSe:Te system to yield potential benefits also in the NPL geometry.

Here, we report for the first time the synthesis and the optical properties of alloyed CdSe_*x*_Te_(1–*x*)_ nanoplatelets. While Te-rich NPLs are shown to exhibit spectroscopic properties similar to both pure CdSe and pure CdTe NPLs (with a modified value of the ‘bulk’ band gap according to the bowing parameter), the optical properties of Se-rich NPLs (*x* > ∼0.7) are shown to be different, appearing to be dominated by states within the gap. Pushing this to the limit of extremely dilute Te-doping of CdSe NPLs, we use single-particle spectroscopy to show the dramatic modification of the photophysics of NPLs even by what seems to be isolated Te substitutional sites within the CdSe host.

## Results and discussion

### Synthesis of three-monolayer thick alloyed NPLs

We first synthesized 3-monolayer (3-ML) thick CdSe_*x*_Te_(1–*x*)_ nanoplatelets while varying the two chalcogenide compositions from *x* = 1 to *x* = 0. Structurally, these NPLs have four cadmium planes in the thickness, intercalated with three anion planes. NPLs are synthesized by a modification of the well-established hot injection procedure developed by Ithurria *et al.* using TOP as the complex reagent for both Se and Te.^1^ To maintain a low monomer concentration during the NPL nucleation and growth, 1 M TOPSe and TOPTe solution precursors were injected at a slow controlled rate by means of a syringe pump. The resulting Se to Te ratio in the final products was in good agreement with the nominal ratio of the precursors introduced into the reaction flask, as corroborated by energy dispersive X-ray (EDX) spectroscopy (Fig. S1 in the ESI†). Further details regarding the synthesis and characterization are provided in the ESI†.

### Band gap bowing in alloyed NPLs

**Fig. 1:**
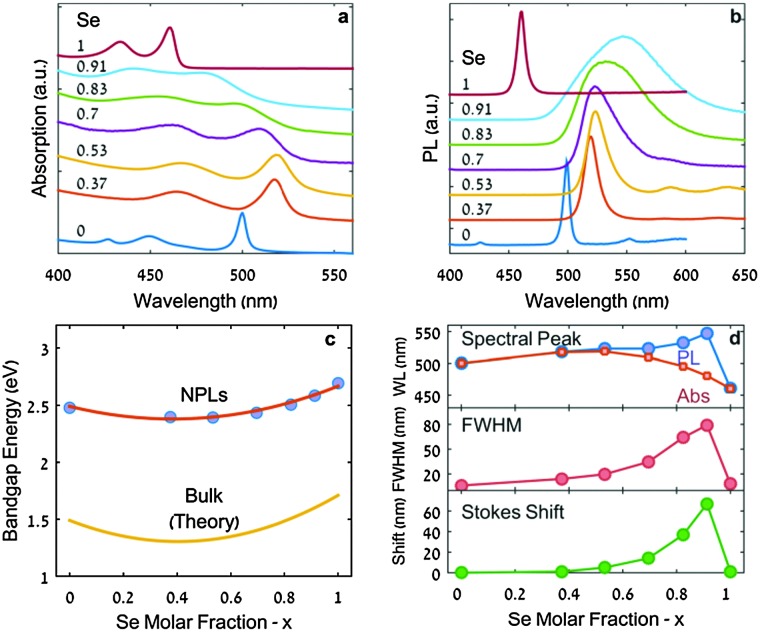
Optical characterization of 3-ML thick CdSe_*x*_Te_(1–*x*)_ NPLs with different compositions. (a and b) Absorption and PL spectra. (c) Experimental curve of band gap energy *versus* Se molar fraction for NPLs (circles) and theoretical curve for bulk CdSe_*x*_Te_(1–*x*)_ (yellow solid line). The solid red line is a fit of the experimental data with the formula given in eqn (1) (*b* = 0.76 eV). (d) From top to bottom: PL peak position alongside the expected bowing dependence of the PL peak, PL FWHM and Stokes shift *versus* Se molar fraction are presented.

The absorption and PL spectra of CdSe_*x*_Te_(1–*x*)_ alloyed NPLs of different Te to Se ratios are presented in Fig. 1a and b, respectively. The absorption spectra of pure CdSe and CdTe exhibit sharp excitonic peaks, characteristic of NPLs.

As the Te content decreases, *i.e.* when *x* increases (bottom to top in Fig. 1a), the absorption peaks gradually red-shift, and broaden slightly up to *x* = 0.5. For *x* > 0.5, we observe a blue shift accompanied by a significant broadening of the peaks. In Fig. 1c we have plotted, indicatively, the edge energy values of the first excitonic peak, for the series of alloyed 3-ML thick CdSe_*x*_Te_(1–*x*)_ NPLs as a function of *x* measured from EDX analysis. The behavior we observe is commensurate with the known band gap bowing in bulk CdSe_*x*_Te_(1–*x*)_ alloys, *i.e.* the parabolic trend of the energy gap with composition.^16,17^

This optical bowing may be described by the following quadratic formula:

1 *E*_g_(*x*) = (1 – *x*)*E*_0_ + *xE*_1_ – *bx*(1 – *x*)

where, *E*_0_ and *E*_1_ are the band gap energies of CdTe and CdSe, respectively, and *b* is the bowing parameter. Fitting the experimental results with eqn (1) (Fig. 1c, red line), we extract a bowing parameter *b* = 0.76 eV. For comparison the band gap bowing for the bulk alloy is also shown in Fig. 1c using the calculated bowing parameter of 0.75 eV (yellow line).^17^ While for spherical CdSeTe quantum dots the non-linearity of the band gap as a function of the anionic composition has been already observed,^20,26^ it is the first time that this phenomenon is demonstrated for colloidal quantum wells.

We assume here that the changes in the absorption spectra are not the result of a change in the number of monolayers in alloyed NPLs. Keeping all other experimental conditions fixed, the variation of the anionic composition is not likely to induce a significant change in thermodynamic conditions required for nucleation of NPLs with different thicknesses. This is particularly so since for single-material NPLs, the variation of the thickness is achieved by introducing great modification in the energetic parameters of the reaction (*e.g.* temperatures or reactivity of precursors).^3^ The agreement between the bowing formula (eqn (1)) and the first exciton energy trend gives further merit to this claim.

The outcome of the syntheses presents a large variance in the lateral dimensions of alloyed NPLs. Its effect on the absorption line position, previously measured as a red-shift of a few nanometers, is negligible in comparison to the tens of nanometers shift observed here for alloyed NPLs.^27,28^

It is worth noting that the absorption characteristics of alloyed NPLs described here are different from those observed in spatially inhomogeneous systems such as CdSe/CdTe core/crown and core/alloyed-crown NPLs. In these systems, exciton transitions, characteristic of two distinct domains, are visible in the absorption spectrum: CdSe core exciton transitions and CdTe crown (or CdSeTe alloyed-crown) exciton transitions.^11–15^ The energies of both transitions match those of the corresponding single-material NPLs with the same number of monolayers. On the other hand, for alloyed NPLs we observed excitonic features characteristic of single-material NPLs, with the exception that the energies of the transitions depend on the anionic composition and shift from pure CdTe NPLs to pure CdSe NPLs.

The maxima of the PL spectra of the alloyed 3-ML thick CdSe_*x*_Te_(1–*x*)_ NPLs (Fig. 1d, blue symbols) red-shift continuously as *x* increases, even for *x* > 0.5. This red shift, which continues up to *x* = 0.9, is accompanied by an appreciable broadening of the emission peak and an increased Stokes shift (Fig. 1d, red and green symbols, respectively). Photoluminescence excitation (PLE) measurements verify that the PL broadening does not arise from the distribution of species in the sample (Fig. S3 in the ESI†). Not only does the PL not follow the first excitonic absorption transition bowing behavior, but there exists an apparently abrupt transition between a 0.1 Te molar ratio concentration and a pure CdSe NPL luminescence peak wavelength. A careful multicomponent fit of the spectra, given in the ESI† (Fig. S4), reveals that none of the components adheres to the band gap bowing trend.

In an analogous bulk alloyed system, ZnSe_*x*_Te_(1–*x*)_, the observed trend of the PL shares some of the features observed here, but significantly differs in others.^29^ There too, at high Te concentrations (*x* < 0.4), the PL closely follows the bowing trend. At lower Te contents, the Stokes shift significantly increases from less than 50 meV up to a value of about 200 meV (for *x* > 0.7) due to the formation of states within the gap associated with ZnTe clusters. Notably, however, in contrast to the continuous redshift of the PL with increasing Se content observed in Fig. 1b, in bulk ZnSe_*x*_Te_(1–*x*)_ PL blueshifts with increasing Te content from the bowing point to the lowest Te content measured. This comparison to ZnSe_*x*_Te_(1–*x*)_ bulk aids in elucidating which effects result from the properties of the bulk alloy and which emerge due to quantum confinement. From this comparison, it appears that Te-associated hole traps are much deeper (relative to the valence band edge) in quantum confined nanoplatelets than in the bulk. This is supported by a recent study of the energetics of small CdTe clusters in CdSe QDs,^30^ where it was shown that isolated Te substitutional sites do not act as hole traps in bulk CdSe, but lead to a deep trap (over 200 mV) in 2.2 nm diameter CdSe QDs.

### Synthesis of Te-doped five monolayer NPLs

The stark difference between the low Te content and pure CdSe samples as well as the apparent effect of quantum confinement on the Stokes shift in the low Te content regime are intriguing features that call for further investigation in order to better characterize the transition between alloyed and doped regimes for CdSeTe 2D nanocrystals.

We therefore set out to fabricate NPLs with low Te contents and to characterize their optical properties. Importantly, at a very low Te content, significant variability between the properties of NPLs within the ensemble is expected due to the stochastic nature of the number of incorporated Te atoms and the distance between them. This variance highlights the need for single-particle emission spectroscopy, as will be outlined below. Since 5-ML thick CdSe NPLs exhibit a higher PL quantum yield, crucial for single particle measurements, it was chosen for the study on dilute Te doping.

The well-established procedure developed by Ithurria *et al.* was used for the synthesis of the 5-ML thick NPLs.^1^ Two variations in the above described synthetic procedure were therefore used to obtain sparsely doped CdSe:Te NPLs. In the first, both Se and Te were introduced in elemental powder form dispersed in ODE (termed Te-powder synthesis). In the second, we used a TOPTe complex and elemental Se precursors dispersed in ODE (termed Te–TOP synthesis). Since under the temperature conditions used for this synthesis Te solubility in ODE is very limited, using the TOPTe complex while leaving the Se precursors in ODE such that only a small amount of TOP was added, allowed for increasing the amount of Te available for reaction without affecting the reaction kinetics.

**Fig. 2:**
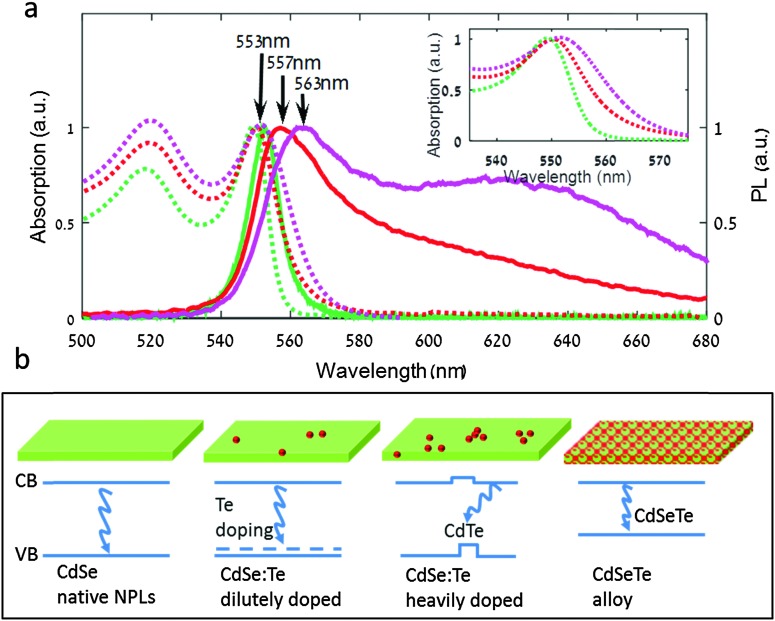
(a) Absorption and photoluminescence spectra measured in the ensemble are denoted by dashed and solid lines, respectively. The undoped CdSe NPLs (green), CdSe:Te NPLs obtained by Te-powder synthesis (red) and CdSe:Te NPLs obtained by Te–TOP synthesis (magenta). Inset: shows the absorption band edge spectra in a higher magnification. (b) A scheme describing the Te atom distribution in the NPLs obtained from different syntheses along with their energy band diagrams, from left to right: NPLs without Te dopant, with low doping density, with high doping density and alloyed NPLs.

Typically, these syntheses yield a NPL size of 10 nm × 25 nm as seen in the TEM images (Fig. S5 in the ESI†). The resulting thickness was determined as 5 monolayers according to the positions of HH and LH transitions measured in the absorption spectra presented in Fig. 2a. The XRD measurements, presented in Fig. S6 of the ESI,† match the bulk CdSe zinc blende crystal structure and show clear similarity to the CdSe NPL XRD data published by Ithurria *et al.* ^3^ The presence of ∼1% Te (atomic concentration) in Te–TOP synthesized NPLs is estimated from EDX studies (see Fig. S7 and S8, ESI†). Although this value is within the instrument detection limit, a careful comparison between EDX spectra in “on NPL” and “off NPL” positions shows a clear preference for the Te presence within the NPLs.

### Ensemble optical spectroscopy of Te-doped NPLs

Fig. 2a presents the absorption and emission spectra of Te-powder and Te–TOP doped and undoped CdSe nanoplatelets. The absorption spectra of the undoped and doped NPLs are almost identical. Yet, the HH transition exhibits a small red shift of 1–3 nm upon doping, which is commensurate with the band gap shift at a doping level of a few percent.^16^ Indeed, some control over the degree of doping by changing the Te precursor's reactivity may be suggested by this slight red-shift of the first exciton peak absorption (HH transition).

In addition, there is a red absorption tail which is shifted to higher wavelengths as measured for the Te–TOP synthesis (inset of Fig. 2a). Such a red tail in the absorption peak has been observed for Te-doped CdSe QDs and is attributed to the HOMO energy levels of the Te dopant contributing to the valance band.^23,24^ Transient absorption measurements (Fig. S9, ESI†) show a rapid transition from induced absorption to bleach within the first 1 ps after the pump excitation. This feature follows the red spectral tail seen in the linear absorption spectrum indicating fast sub-ps cooling dynamics into long lived Te states.

While the absorption spectra strengthen the observation that the different syntheses lead to NPL formation, it only shows extremely minor modifications due to Te incorporation within the NPLs. However, major differences appear in the PL spectra of Te-doped NPLs against that of native NPLs. For the Te-powder synthesized NPLs, a pronounced red tail is measured, whereas for the Te–TOP synthesized NPLs a second wide peak centered at around ∼630 nm emerges. Indeed, such sharp changes agree with the drastic difference between the PL spectra of CdSe and CdSe_0.91_Te_0.09_ NPLs (Fig. 1a and b) and show that Te doping indeed strongly affects the luminescence rather than the absorption spectrum.

A variety of charge traps either by surface states or defects due to doping can generate wide spectra similar to the one observed in Fig. 1a by simply decreasing the band-edge emission while introducing some in-gap states which fluoresce weakly. To examine if this is indeed the case for Te-doped NPLs, we measured the fluorescence quantum yield (QY) of NPLs obtained from the different syntheses. The QY was measured to be 28% and 40% for Te-powder and Te–TOP synthesized NPLs, respectively, while similar values of 16–35% were measured for undoped NPLs. Variations in the measured QY of undoped NPLs are probably due to differences in ligand surface passivation that occur from slight differences in the cleaning procedure employed. The relatively high QY values of the doped NPLs (which are comparable and even slightly higher than those of undoped ones) indicate that their PL spectral broadening is not generated by surface trap emission, but rather by actual substitutional doping.

Furthermore, since Te is present in the solution throughout the entire synthesizing reaction it seems likely that it is incorporated within the CdSe lattice as the NPLs grow. Segregation to the surface is unlikely due to the extremely slow expected solid state diffusion rate of Te within CdSe at the reaction temperature (240 °C).^31,32^ Interstitial doping of Te within the CdSe matrix seems unlikely for two reasons. First, the large ionic radius of Te^2–^ is larger by ∼10% than that of a Se^2–^ ion and will therefore impose strain in any interstitial site. In addition, such a defect would need to have an oppositely charged partner. A substantial strain field, created by the pair, close to a free standing surface causes this state to be thermodynamically unfavorable. We therefore consider the incorporation of Te in these structures, as in the alloyed NPLs, to be in substitutional sites only.

### Single-particle optical spectroscopy of Te-doped NPLs

It appears evident that the PL spectra of the NPLs obtained from both Te-incorporated syntheses are comprised of two overlapping emission peaks suggesting two different species schematically illustrated in Fig. 2b: lightly Te-doped NPLs (II) and NPLs with at least one CdTe cluster (*i.e.* “heavily doped”) (III). The term ‘CdTe cluster’ refers, in this context, to two or more Te atoms occupying neighboring chalcogenide sites in the CdSe lattice.

The evidence of a multi-species synthesis product, obscuring spectroscopic features at the ensemble level, shows the importance of performing single-particle spectroscopy measurements when characterizing these low doping level nanocrystals.

**Fig. 3:**
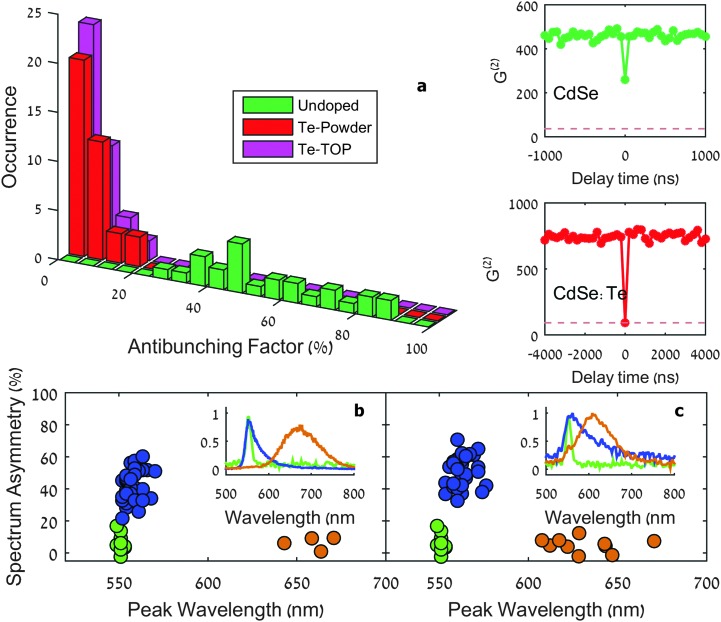
Distinguishing the synthesis outcome using single-particle spectroscopy. (a) Histogram of the antibunching factor for the undoped NPLs (green), Te-powder doped NPLs (red) and Te–TOP doped NPLs (magenta). The insets present two representative *G* ^(2)^ curves for undoped (top) and doped (bottom) NPLs. The dashed lines in the insets represent the *G* ^(2)^ dark level used in eqn (2). (b and c) Scatter plots of the spectrum peak wavelength and peak asymmetry of single NPLs obtained from Te-powder synthesis (b) and Te–TOP synthesis (c). In both figures, the lightly doped NPLs (blue) and heavily doped NPLs (orange) are plotted together with the undoped (native) NPLs (green) for comparison. The asymmetry is defined as the difference between the area under the red side and the blue side of the PL peak divided by the total area of the PL. Representative spectra of individual NPLs are given in the inset of each figure following the same color code as (b) and (c).

Single-nanocrystal spectra and a time-resolved single-photon correlation measured in a Hanbury-Brown and Twiss (HBT) setup were collected consecutively for each single particle. The most striking difference between doped and undoped NPLs, as revealed from all single-particle measurements, is the antibunching feature. Eqn (2) defines the antibunching factor (ABF) which quantifies to what extent a nanocrystal can be considered as a single-photon emitter.2
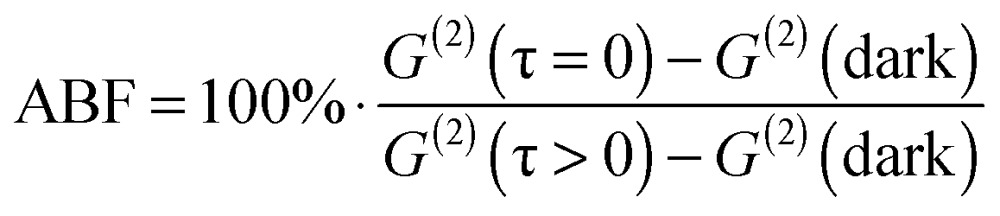
 where, *G* ^(2)^(*τ*) is the number of photon pairs detected within a certain delay between their arrival times measured in terms of the number of pulses (see the insets of Fig. 3a). *G* ^(2)^ (dark) refers to an estimated number of detection coincidences in which at least one detection is the result of the detector dark current and *G* ^(2)^(*τ* < 0) denotes an average photon pair number over the pulse difference range between 3 and 30. In this scale, 0% ABF is indicative of exclusively single-photon emission, while 100% ABF means no suppression of photon pair fluorescence. Typically, single QDs have been shown to exhibit a low probability of simultaneous multiple photon emission due to the rapid non-radiative Auger decay of multiple-excited states; corresponding to a value of ABF close to zero.^33–35^ However, in the case of NPLs, previous studies have reported a non-negligible biexciton quantum yield (BXQY) leading to appreciably larger values of the ABF.^15^ This feature was attributed to the extended size of the lateral dimension that results in a continuous density of states for the charges, and reduces the non-radiative Auger recombination rate. Our results for the undoped NPL sample indeed found a distribution of ABF values for blinking, apparently single NPLs, ranging from about 30% to 90%. Fig. 3a shows an ABF histogram of single NPLs for the undoped (green), doped Te-powder (red) and doped Te–TOP (magenta) NPL samples. One can see that while for undoped NPLs the ABF is higher than 30% and reaches values as high as 90%, for NPLs obtained from both doping-based syntheses the ABF does not exceed 10% for the majority of NPLs. These results match intuition gained by previous research on Te-doped QDs, in which it was indirectly inferred that the biexciton Auger lifetime was reduced by the incorporation of a Te dopant.^36,37^ This reduction was explained to stem from the Te dopant acting as a deep hole-trap, increasing the local hole–hole Coulomb interaction and therefore increasing the rates of Auger recombination.^30,37^ In a similar manner, Tessier *et al.* have shown that for CdSe/CdS core/crown NPLs,^15^ decreasing the CdSe core size yielded a lower ABF in single-particle emission. Similarly to the mechanism suggested here, the increased probability for Auger recombination was explained by the strong confinement of holes within the small CdSe cores.

While the ABF measurements clearly show a drastic effect of Te incorporation within CdSe NPLs that cannot be revealed in ensemble measurements, they do not address the question of inhomogeneity within the doped NPLs, as the ABF has a continuous distribution for Te-incorporated NPLs. We have therefore measured, alongside the HBT measurements, the PL spectrum of each of the single NPLs analyzed. Three typical single NPL emission spectra are given in the insets of Fig. 3b and c. While the spectra of single undoped NPLs (green) resemble their ensemble spectra (Fig. 2a), featuring a narrow peak centered at ∼555 nm, the Te-incorporated samples display two distinct types of spectra: a ∼560 nm peaked spectrum with an asymmetric red tail (blue) and a second type with a symmetric wide peak centered at ∼650 nm (orange). To analyze the difference between these two types of spectra Fig. 3b and c present a scatter plot of the spectral peak of single NPLs *versus* peak asymmetry for the Te-powder and Te–TOP samples, respectively (see the ESI† for the definition of peak asymmetry). Native NPLs (Fig. 3b and c green circles), shown for comparison, exhibit symmetric spectra peaking at 555 nm, whereas for both Te-incorporating syntheses two groups of NPL species are evident (Fig. 3b and c blue and orange circles). The first group has highly asymmetric spectra with peaks centered at 555–560 nm, whereas the second displays symmetric spectra with peak emission widely distributed between 600 and 700 nm.

The first species, displaying heavy tailed spectra, resembles the results obtained for Te-doped CdSe QDs which were shown to include only a single Te emitting center, in which the PL emission was red-shifted and developed a red tail.^23,30^ While the line shape of the red tail is similar to that of QDs, the red shift of the peak is much more pronounced in 3D nanocrystals compared to the ∼5 nm shift measured here for NPLs.^32^

While isolated dopant atoms (Fig. 2b, second diagram) may explain the spectra of the first species, the second, with a broadened symmetric red shifted emission, can be attributed to NPLs with at least one CdTe cluster (Fig. 2b, third diagram). Such a cluster leads to a distribution of trap states. Indeed similar single particle spectra were observed for Te-doped QDs.^23^ In addition, it was shown that for core/crown CdTe/CdSe NPLs with small cores the absorption is dominated by the large CdSe crown, while the emission is dominated by the low energy type-II spatially indirect exciton.^11,13^

The main difference, with respect to this analysis, between the NPLs obtained from the two syntheses is the higher occurrence of the red peaked species in the doped Te–TOP sample, suggesting that these are indeed the products of higher Te concentration NPLs, which result in the formation of CdTe clusters.

Notably, PLE measurements at all emission wavelengths match the linear absorption spectra of NPLs (for example, see Fig. S10, ESI†), indicating that the absorption originates from the “bulk” of the CdSe matrix, while the emission originates from the Te states. In addition, this serves as direct proof that indeed all observed emissions are due to the excitation of doped NPLs and not other species such as CdTe or alloyed NPLs.

### Photoluminescence transients in alloyed and doped NPLs

To further elucidate the transition from doped to alloyed NPLs, we explore the differences in the temporal transients of luminescence. While for the alloyed samples ensemble (in solution) measurements are sufficient to extract the dynamics, in the case of Te-doped NPLs the reaction products are mixtures of two species and therefore have to be studied as single particles. In order to compare the outcome of ensemble and single-particle measurements, we average the transients of over ∼30 NPLs of the lightly doped species and ∼10 NPLs of the heavily doped species. To overcome the issue of excessive blinking of NPLs (like QDs) on a substrate, we post-select detections that were measured only during the ‘on’ state emission by using a threshold (see ESI†).

**Fig. 4:**
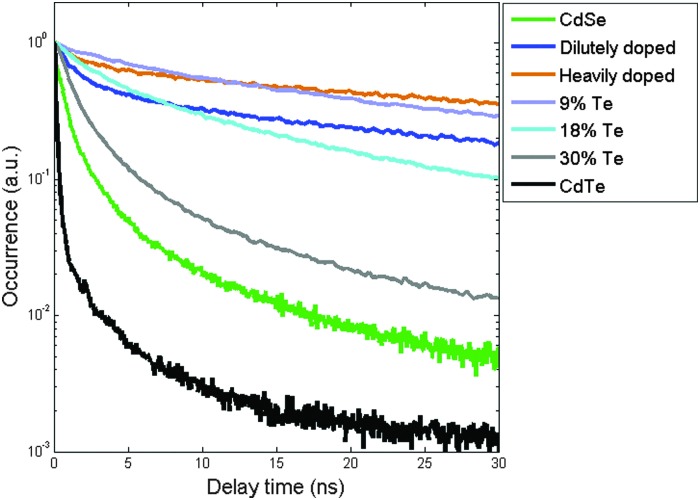
PL transients of pure CdSe (green) and CdTe (black) NPLs are shown together with similar curves measured for alloyed NPLs with different Te compositions (9% Te purple, 18% Te cyan and 30% Te grey). The lifetime curves of lightly doped and heavily doped NPLs, derived from averaging single-particle luminescence are shown in blue and orange, respectively.

In the PL transient of both native CdTe (black) and CdSe (green) NPLs, shown in Fig. 4, the most prominent component is a fast one with a lifetime of ∼1 ns and ∼2 ns, respectively. As the concentration level decreases below 30% Te, a long lifetime component (∼50 ns) becomes more dominant. Finally, for the 9% Te sample, the lifetime curve is composed entirely of a single exponent. At this point, the trend of lifetime increase is inverted and for heavily Te-doped species the fast lifetime component re-appears, becoming even more visible in the case of lightly doped NPLs.

A similar trend of an emergent long lifetime component was shown in Te-doped CdSe QDs going from pure CdSe QDs to 5% Te content nanoparticles.^23^ Furthermore, a full systematic lifetime study performed for the bulk ZnSe_*x*_Te_(1–*x*)_ system shows initially an increase of the average lifetime with the increased Te concentration up to 10% followed by a return to an emission lifetime characteristic of pure ZnSe at higher Te concentrations.^38^ In addition, the stretched exponential behavior of the lifetimes in this system was explained by the hopping-transport model where the transfer of excitons from shallow to deep Te localized states provide multi decay paths.^39^ Such multi-exponential transients are more difficult to analyze in this system since pure CdSe and CdTe NPLs themselves present multiple time scales in the PL transients. However, qualitatively, we can see in Fig. 4 that higher Te concentration curves (*e.g.* 18% and 30%) appear to be “smoother”, that is they cannot be fit by just one or two exponentials, but hint at a distribution of recombination rates as the model described in ref. 39 predicts.

For the ZnSe_*x*_Te_(1–*x*)_ bulk system in the low Te concentration side, as the Te content increases the hybridization with valence band edge states results in the decrease of the PL lifetime and linewidth. In contrast, in the low Se content regime, there is no change since Se levels lie above the top of the valance band in ZnTe.^40^

## Conclusions

In conclusion, we have shown the complete optical transition from lightly Te-doped CdSe NPLs to CdSe_*x*_Te_1–*x*_ alloyed NPLs. We have demonstrated the synthesis of Te-doped CdSe NPLs and an extensive control over the synthesis of alloyed NPLs. At Te concentrations higher than ∼30%, the CdSe_*x*_Te_(1–*x*)_ NPLs show a bowing phenomenon of the band gap for increased concentrations of dopant analogous to an alloyed bulk material. However, the PL peak wavelength does not follow the same trend at lower Te concentrations and instead redshifts with decreasing Te contents, creating a sharp transition from pure CdSe NPLs. This is due to the presence of Te substitutional sites which act as deep hole traps and dominate the luminescence spectrum. Reducing the Te concentration towards the dilute doping regime reveals a stepwise drastic change in the PL spectra, which we associate with a transition from small CdTe clusters to isolated Te substitutional sites. Furthermore, lifetime measurements show a dramatic increase from the pure CdSe NPL case to the doped ones associated with a Te deep hole trap. At increased Te contents (above ∼10%) the lifetime gradually decreases back to values similar to the single material nanoplates, indicating the absence of trap states within the gap. The stark difference between pure and doped NPLs is also shown in photon antibunching measurements, where the probability of emitting photon pairs is highly reduced in doped NPLs. These findings manifest the stepwise transition from pure CdSe NPLs to Te-doped NPLs and then a continuous gradual transition for CdSe_*x*_Te_(1–*x*)_ alloyed NPLs with increased Te content.

## Materials and methods

### Alloyed NPL synthesis

A three-neck flask was charged with 120 mg of Cd(Myr)_2_ (0.21 mmol) in 12 mL of ODE. The mixture was degassed under vacuum at RT for 30 min. Then, under Ar flux, the temperature was increased to 240 °C and 60 mg of Cd(OAc)_2_·2H_2_O (0.23 mmol) and 160 mg of Cd(prop)_2_ (0.62 mmol) were added. After 1 min, a solution composed of 150 μL of a variable ratio of TOPTe 1 M and TOPSe 1 M, and 200 μL of OA (0.63 mmol) in 2 mL of ODE were added using a syringe pump. The injection rate was fixed at 8 mL h^–1^. After 30 min from the onset of the injection, the heating mantle was removed and the mixture cooled down. At 100 °C, 1 mL of OA was added. At room temperature, 15 mL of hexane and 10 mL of EtOH were added to the mixture. NPLs were precipitated by centrifugation at 5000 rpm for 5 min and the selective precipitation was repeated 2 times replacing hexane with toluene. Finally, NPLs precipitated were re-dissolved in 5 mL of toluene.

To synthesize alloyed CdSe_*x*_Te_(1–*x*)_ NPLs with variable compositions, we performed several experiments where the nominal molar ratio Se : Te was varied as follows: 100 : 0, 90 : 10, 80 : 20, 66.6 : 33.4, 50 : 50, 33.4 : 66.6 and 0 : 100.

### Doped NPL synthesis

A mixture of 0.3 mmol cadmium myristate (Cd(Myr)_2_) and 15 mL of octadecene (ODE) were put in a flask and degassed under vacuum at 100 °C for 1 h. The temperature was then increased to 240 °C under argon flow. A mixture of selenium (Se) and tellurium (Te) consisting of 0.075 mmol Se and 0.075 mmol Te dispersed in 1 mL of ODE was rapidly injected at 240 °C. Alternatively, a Se/Te stock consisting of 0.135 mmol Se dispersed in 1 mL of ODE mixed with 0.1 mL of TOPTe stock (0.15 mmol Te and 0.15 mmol trioctylphosphine (TOP) dissolved in 1 mL ODE under moderate heat until a clear greenish solution was obtained) was used. After 20 s, 0.3 mmol of cadmium acetate (Cd(OAc)_2_·1H_2_O) was dispensed into the flask. The synthesis was monitored by absorption and photoluminescence measurements using a UV-VIS spectrophotometer (V-670, JASCO) and a fluorimeter (USB4000, Ocean Optics), respectively. After ∼10 min, 4 mL of oleic acid (OA) was injected into the flask and the growth was quenched by lowering the temperature. The NPLs were then precipitated out from chloroform and redispersed in hexane.

### Single particle spectroscopy

Highly dilute solutions (∼10^–12^ M NPL concentration) of doped and pure 5-ML thick NPLs in hexane were spin coated onto a glass cover slip. The sample was measured using an optical setup built around a commercial microscope (Axiovert 200, Zeiss). 473 nm 100 ps laser pulses at 5–20 MHz repetition rate (EPL 470, Edinbrough Instruments) were tightly focused through a 1.3 N.A. objective lens (Zeiss) to a diffraction limited spot. PL light was collected through the same objective lens passing through a dichroic mirror (488LP Semrock) and a long-pass dielectric filter (488LP Semrock). Light was then steered into one of the two ports. The first was a Hanbury-Brown and Twiss setup in which light, including the entire PL spectrum, was coupled into a split-fiber coupled to two avalanche photo diodes (APDs) (Perkin Elmer SPCM). The APD signal was routed into a timing module (Picoquant, Hydraharp 400) and was analyzed digitally using MATLAB software. The second port was a fiber coupled spectrometer (Princeton Instruments, Acton SP2300i) equipped with a 300 g mm^–1^ grating whose output was measured using a cooled CCD (Pixis, Princeton Instruments).

Approximately 100 single particles of the undoped, doped Te-powder and Te–TOP samples were examined altogether.
