# Supplementary material for: From dilute isovalent substitution to alloying in CdSeTe nanoplatelets
Source: Phys Chem Chem Phys. 2016 May 5;18(22):15295–303. doi: 10.1039/c6cp01177b (PMC5040066; doi:10.1039/c6cp01177b)
Supplement: Supplementary file 1 [file CP-018-C6CP01177B-s001.pdf]

Electronic Supplementary Material (ESI) for Physical Chemistry Chemical Physics.
This journal is © the Owner Societies 2016

Supporting Information

From Dilute Isovalent Substitution to Alloying in CdSeTe Nanoplatelets

Ron Tenne[1], Silvia Pedetti[2,3,] Miri Kazes[1], Sandrine Ithurria[2], Lothar Houben[4], Brice Nadal[3], Dan Oron[1], Benoit Dubertret[2]

[1] *Department of Physics of Complex Systems, Weizmann Institute of Science, 76100 Rehovot, Israel*

[2] *ESPCI ParisTech, PSL Research University; CNRS; Sorbonne Universités, UPMC Univ. Paris 6; LPEM, 10 rue Vauquelin, F-75231 Paris Cedex 5, France*

[3] *Nexdot, 10 Rue Vauquelin, 75005 Paris, France*

[4] *Department of Chemical Research Support, Weizmann Institute of Science, 76100 Rehovot, Israel*

EDX Analysis of Alloyed NPLs

NPLs solutions dispersed in hexane were deposited on carbon film dropwise to form a thin film. Samples were dried under vacuum overnight and then analyzed. EDX measurements were done under 15V acceleration voltage during a 120sec acquisition time. Data analysis was performed using a FEI Magella according to $L\alpha$ x-ray lines of Cd,Se and Te. Figure S5 compares the nominal concentrations in the syntheses with the EDX measured. Concentration of Se,Te here is defined as the atomic portion of the chalcogenide.

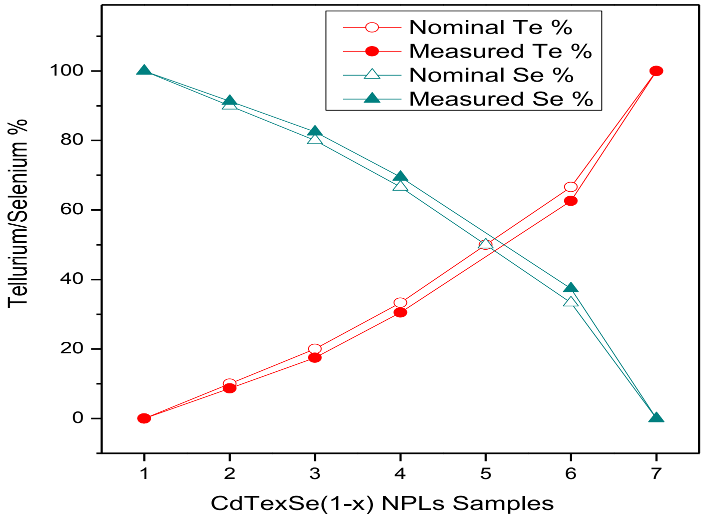

Figure S1: Te,Se content for various syntheses (numbered 1-7). Full symbols show the EDX results while empty symbols show the nominal concentrations as introduced into the reaction flask.

Transmission Electron Micrographs of alloyed CdSexTe(1-x) NPLs

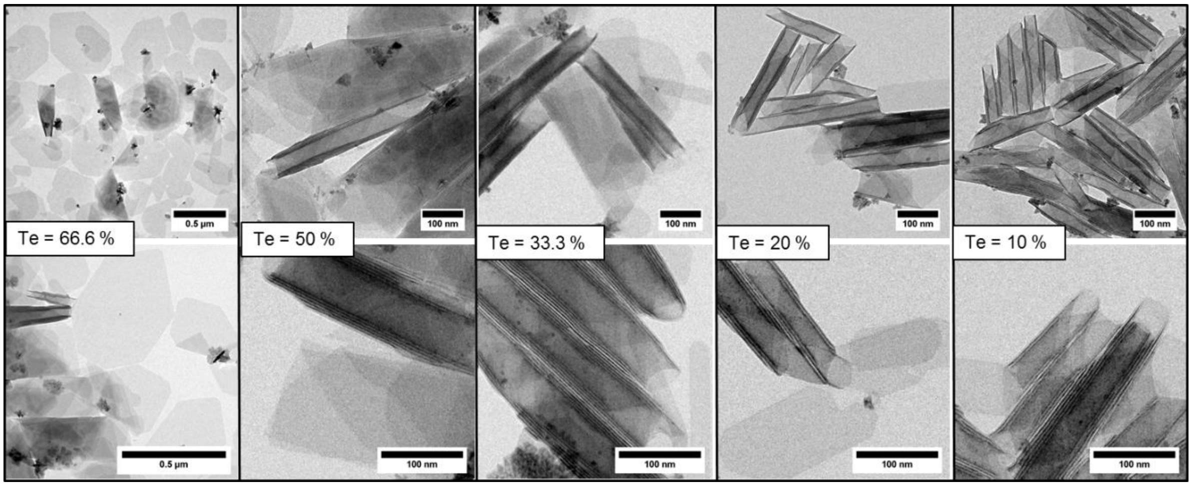

Figure S2: TEM images of $CdSe_xTe_{(1-x)}$ NPLs with nominal a Te concentration (as introduced into the reaction flask) of 66.6 %, 50 %, 33.3 %, 20 % and 10 %.

Photoluminescence Excitation (PLE) of alloyed NPLs

Photoluminescence Excitation (PLE) Measurements were acquired with Horiba Jobin-Yvon Fluoromax-3 spectrometer. NPLs were dispersed in hexane. The excitation wavelength was fixed at max wavelength of the PL and max + 1/2 and max -1/2. Even though the photoluminescence of alloyed NPLs with Te percent content of 17% and 9%, is characterized by a FWHM of up to 80 nm, the PL excitation spectra at different emission wavelengths show the same features for different detection wavelengths.

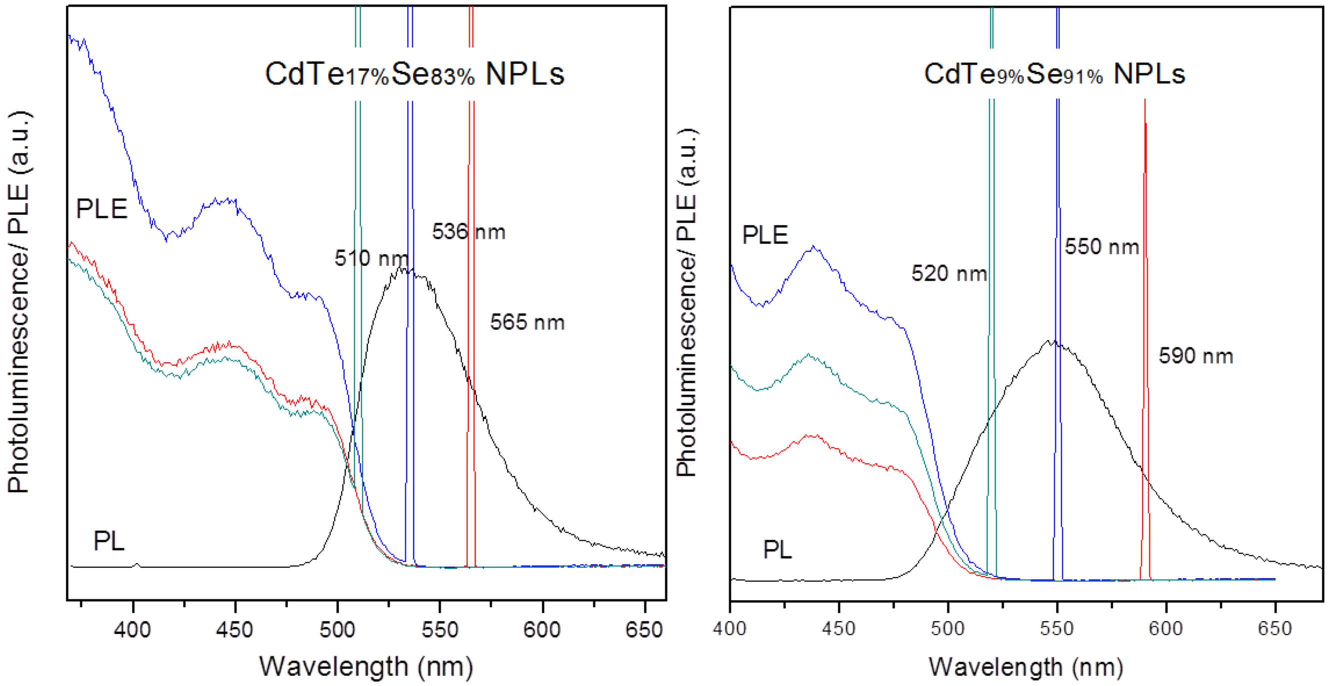

Figure S3: PLE spectra at three emission wavelengths of CdTeSe NPLs with Te percent content of 17% and 9%, (a) and (b) respectively.

Fit Analysis for the Photoluminescence of Alloyed $CdSe_xTe_{(1-x)}$ NPLs

Figure 1d shows the drastic change in the PL peak position between a pure CdSe sample with a peak at around 461nm to 547nm for a sample with only 9% Te content. While the photoluminescence spectra of pure CdSe and CdTe NPLs present a single sharp peak per sample, alloyed samples present wider and asymmetric shaped spectra (Figure 1). In order to exclude the appearance of a drastic change of spectra merely due to an inhomogeneous outcome of the synthesis fit the spectra to a multi-component one and check the behavior of the different components.

To do so we fit each of the alloyed NPLs PL spectra with a sum of 2 or 3 Gaussian functions (with respect to PL peak energy) using a least mean squares approach. Figure S4 shows the peak position of the different components *versus* the Se content in the NPL synthesis. Here the orange curve corresponds to the longest wavelength component, the blue to the shortest wavelength component and the red to the middle wavelength component. At points in which less than three Gaussians were employed the curves intersect. The absorption spectrum peak is shown for comparison in violet.

It is clearly seen that even if we take into account only the highest energy component there is no substantial bowing in the photoluminescence as there is in the absorption. Even for this component the PL peak shifts by >250meV with the substitution of 9% of the Se with Te.

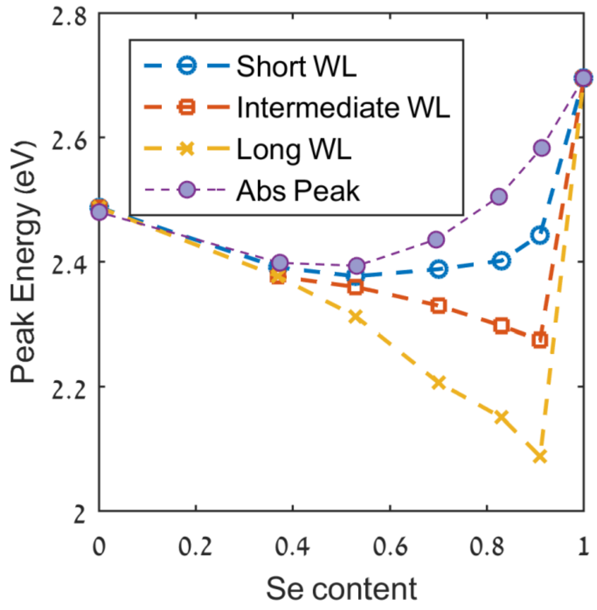

Figure S4: PL spectra peak position of the highest energy (blue), intermediate energy (red) and low energy (orange) fit components of the PL spectra *versus* the Se content in the sample. Absorption spectra peaks (violet) are given for comparison.

Transmission Electron Micrographs of Te-doped CdSe NPLs

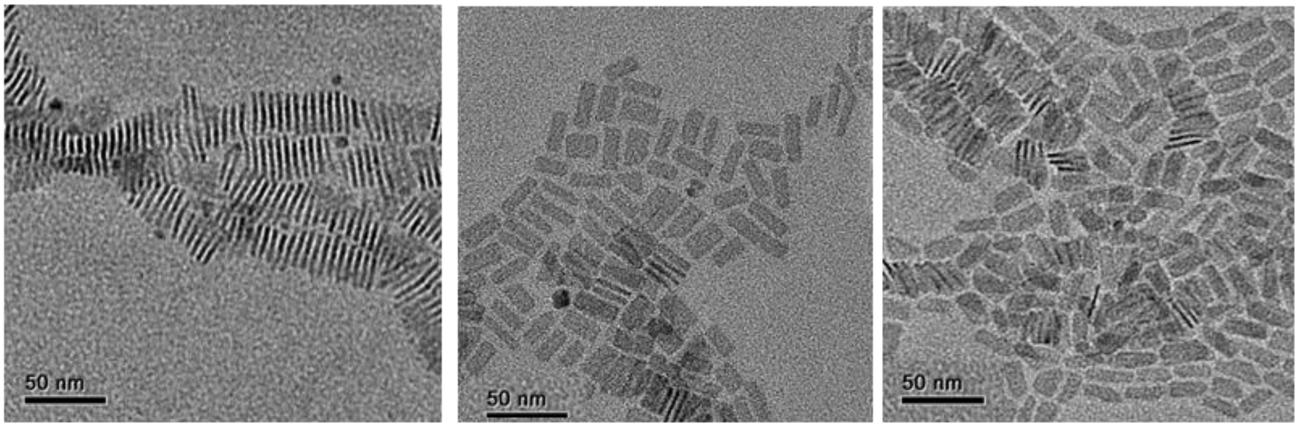

Figure S5: TEM images of undoped CdSe NPLs (left) and Te doped NPLs by synthesis route 1 (middle) and by route 2 (right). All showing NPLs of similar lateral dimensions of 10nm x 25nm. The TEM images were taken at 120 kV, using a Philips, CM-120 TEM instrument.

XRD powder diffraction of Te-doped CdSe NPLs

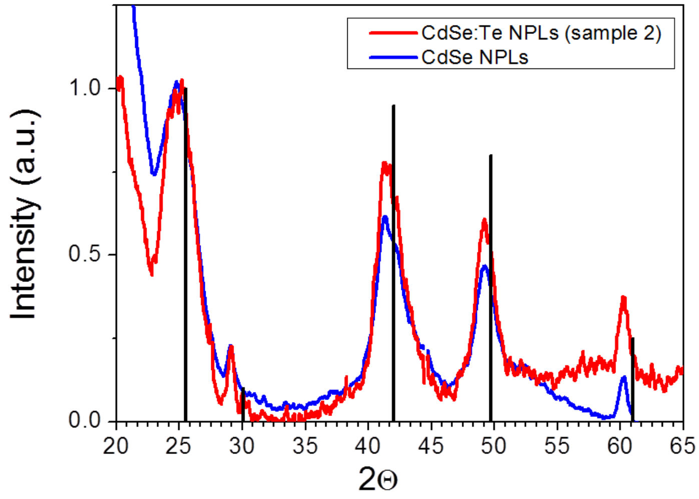

Figure S**6**: XRD powder diffractograms of the Te doped and undoped NPLs (red and blue respectively) both showing the peaks corresponding to a zinc blende bulk CdSe (in black).

Atomic-resolution STEM images and EDX maps for doped NPLs

STEM images and EDX maps were recorded at an accelerating voltage of 80 kV in a probe aberration-corrected FEI Titan G2 60-200 ChemiSTEM microscope equipped with a high-brightness FEG. The samples were treated in an Ar/O plasma to prevent from hydrocarbon contamination during TEM observation. HAADF images were recorded with a Fischione Model 3000 detector with a semi-convergence angle of 23.8 mrad, a probe current of 40 pA, and an inner collection angle of 99.0 mrad. EDX spectrum image data were obtained with a Bruker Super-X four-segment SDD detector with a probe semi-convergence angle of 23.8 mrad and 35.9 mrad, a beam current of 70 pA and 124 pA, respectively, and a total recording time of 600 s. Quantitative maps were calculated with the Bruker Esprit software, through background subtraction and spectrum deconvolution.

Fig S6 presents an EDX spectrum image of Te-TOP NPLs that compares the measurement on NPL and on the carbon support aside from the NPL. Part (a) shows a superposition of the simultaneously acquired HAADF signal and the net count map for Cd (blue), Se (green) and Te (red). Part (b) is a summary of quantification results obtained for the sum spectra of two regions, either on the NPL or aside on the support grid. Raw data and deconvolution of ranges in the EDS sum spectra of the NPL region in (c) shows the presence of the Te M-series as a shoulder to the O K line and the presence of the Te L-series on top of the Cd L-series.

The isolated Te map on the right hand side indicates that almost all Te EDS counts are generated on the NPLs. Inspection of the data tables in (b) shows that the Te L-series net count rate measured aside the NPL is considerably lower than the net count rate measured on the NPL, by a factor of four when normalized to the same area. The Te-L net count rate aside the NPL is the lower threshold for the uncertainty of the net counts measured on the NPL. The corresponding lower threshold for the uncertainty of the Te concentration measured on the NPL is approximately 0.4 %. To approach this uncertainty of the Te concentration including the shot noise and background noise all measurements were done with the long exposure time of several minutes and a quantification was done on a larger region of interest, *i.e.* large number of spectra. Our estimate of a detection threshold of 1 % including systematic and unsystematic noise is conservative. For the NPL region in Fig S6 we can conclude that there is a 1 % concentration of Te in the NPL above the detection limit.

Fig.S7 presents EDX elemental mapping of another area containing a few Te-TOP NPLs (a) alongside a HAADF image of the same area (b). A clear increase of Cd (blue) and Se (green) concentration appears in the NPLs region together with a slight increase in Te (red) concentration. Panel (c) shows the spectra analysis for three sub-areas of (a) (numbered green markers). Analysis of the NPL region (sub-area 3) indeed shows an increased signal in the Te L-series line corresponding to an estimate of 2.9 % atomic concentration of Te with the nanocrystal. This value is well above the detection limit of the instrument (1%), the 2.1 % Te concentration found for the entire area (sub-area number 1) suggests an estimate of at least ~1 % Te concentration within the NPLs. Sub-area 2 included a small cluster aside the NPL that contained either non-reacted elemental tellurium or tellurium oxide. This region was used to gauge the sensitivity for the low count rate of EDS Te-L signal generated from a small cluster of atoms and to exclude the possible overlap with the Cd L-series as the origin for these counts.

Unfortunately, the low signal to noise ratio of the Te line signal generates a noisy image limiting the spatial resolution of features within the NPL. For this reason we cannot be sure whether clustering of CdTe within the NPL occurs.

a.

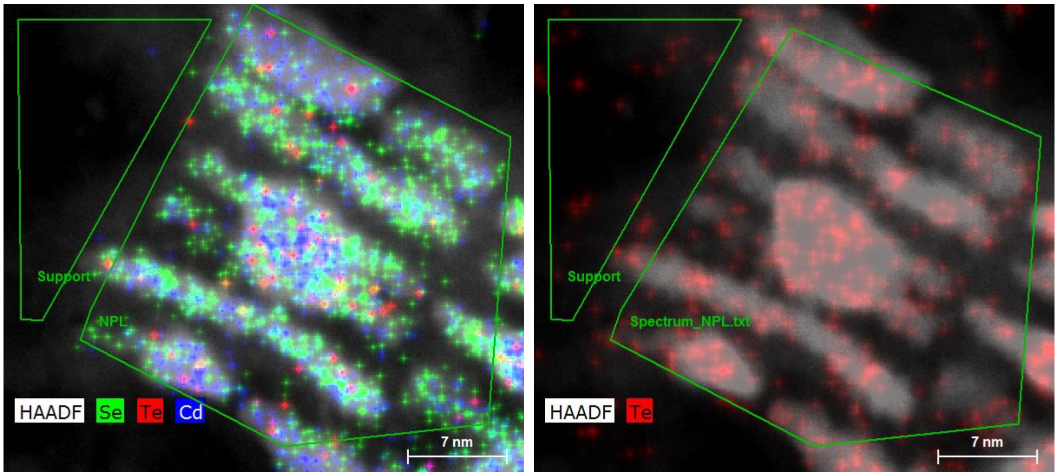

b.

Support (number of spectra: 10689)

El	AN	Series	Net	unn. C [wt.%]	norm. C [wt.%]	Atom. C [at.%]	Error (1 Sigma) [wt.%]
C	6	K-series	1624	84.67	84.67	91.90	3.32
O	8	K-series	354	8.34	8.34	6.80	0.53
Cu	29	K-series	149	5.55	5.55	1.14	0.54
Cd	48	L-series	21	0.80	0.80	0.09	0.27
Te	52	L-series	10	0.50	0.50	0.05	0.23
Se	34	L-series	4	0.13	0.13	0.02	0.10

NPL (number of spectra: 42517)

El	AN	Series	Net	unn. C [wt.%]	norm. C [wt.%]	Atom. C [at.%]	Error (1 Sigma) [wt.%]
Cd	48	L-series	5634	68.41	68.41	60.97	6.97
Se	34	L-series	3200	29.42	29.42	37.32	3.03
Te	52	L-series	164	2.17	2.17	1.71	0.34
Cu	29	K-series	4334	-	-	-	-
O	8	K-series	2682	-	-	-	-
C	6	K-series	12778	-	-	-	-

c.

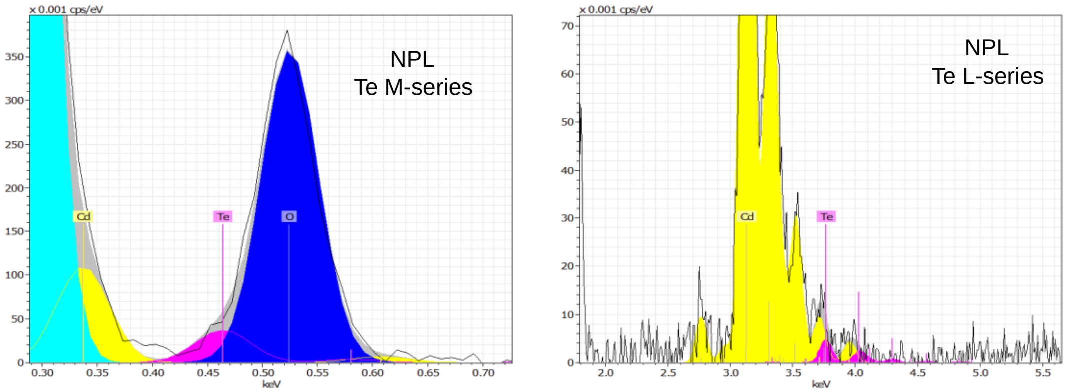

Fig. S7: EDS spectrum image data of an assembly of CdSe:Te NPLs synthesized with the Te-TOP procedure. (a) Chemical maps of Cd, Se and Te obtained after background subtraction and spectrum deconvolution. (b) Quantification of the chemical composition for the sum of spectra of two regions of interest, on the NPLs and on the support aside. The corresponding regions of interest are marked in part (a). (c) Spectrum deconvolution for the NPL sum spectrum.

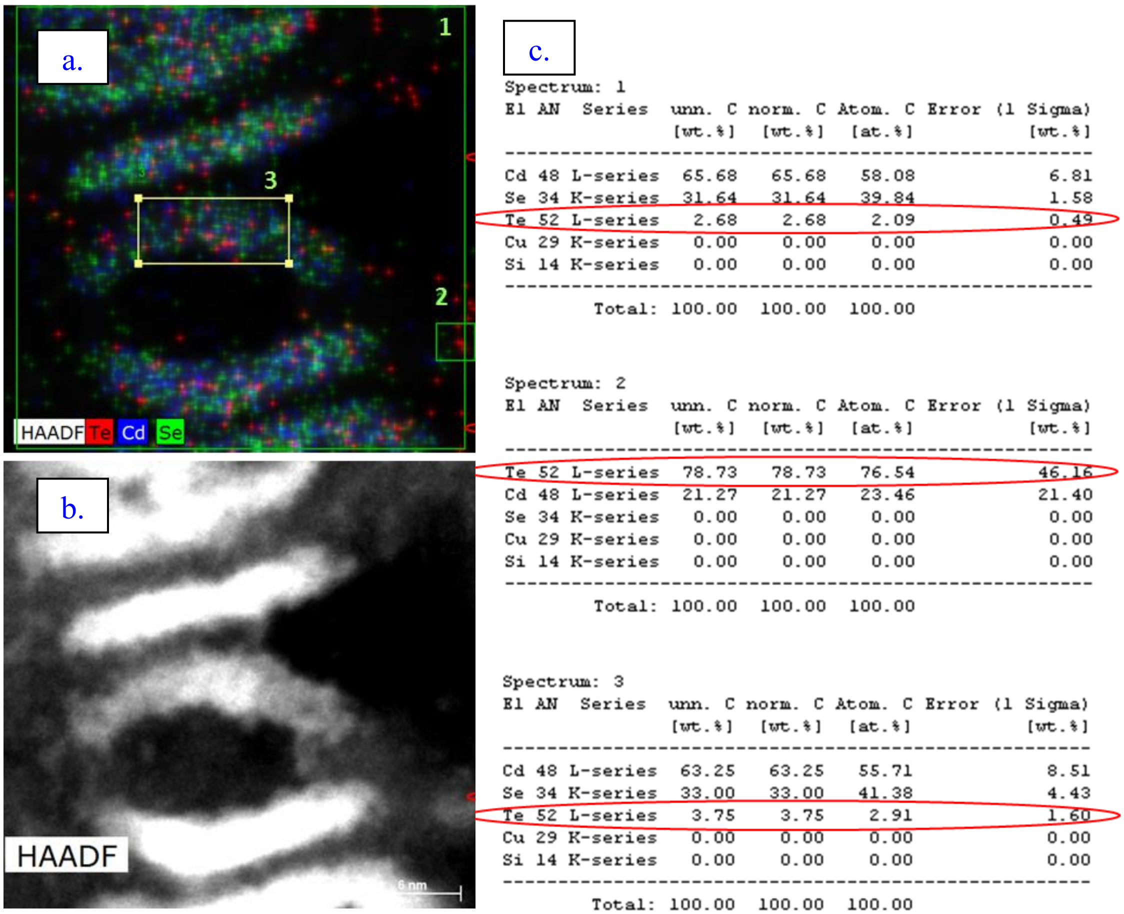

c.

Spectrum: 1

El	AN	Series	unn. C [wt.%]	norm. C [wt.%]	Atom. C [at.%]	Error (1 Sigma) [wt.%]
Cd	48	L-series	65.68	65.68	58.08	6.81
Se	34	K-series	31.64	31.64	39.84	1.58
Te	52	L-series	2.68	2.68	2.09	0.49
Cu	29	K-series	0.00	0.00	0.00	0.00
Si	14	K-series	0.00	0.00	0.00	0.00
		Total:	100.00	100.00	100.00	

Spectrum: 2

El	AN	Series	unn. C [wt.%]	norm. C [wt.%]	Atom. C [at.%]	Error (1 Sigma) [wt.%]
Te	52	L-series	78.73	78.73	76.54	46.16
Cd	48	L-series	21.27	21.27	23.46	21.40
Se	34	K-series	0.00	0.00	0.00	0.00
Cu	29	K-series	0.00	0.00	0.00	0.00
Si	14	K-series	0.00	0.00	0.00	0.00
		Total:	100.00	100.00	100.00	

Spectrum: 3

El	AN	Series	unn. C [wt.%]	norm. C [wt.%]	Atom. C [at.%]	Error (1 Sigma) [wt.%]
Cd	48	L-series	63.25	63.25	55.71	8.51
Se	34	K-series	33.00	33.00	41.38	4.43
Te	52	L-series	3.75	3.75	2.91	1.60
Cu	29	K-series	0.00	0.00	0.00	0.00
Si	14	K-series	0.00	0.00	0.00	0.00
		Total:	100.00	100.00	100.00	

Figure S8: HR-STEM and EDX measurement of CdSe:Te NPLs synthesized using the Te-TOP procedure. An elemental map produced from EDX spectra is shown in (a) presenting the concentration of Cd (blue), Se (green) and Te (red). The increased abundance of Cd,Se and Te within the NPLs is clear from comparison to the HAADF image of the same area given in (b). Quantitative analysis of the spectra over three sub-areas, marked in (a) by green numbered rectangles, are shown in (c).

Transient absorption measurements

Transient absorption measurements were performed using a 120 fs pump pulse at 400 nm with 250Hz repetition rate and a beam diameter of about 500 μm. The NPLs in their excited state is

then probed with a broad band "white" pulse at different delay times after the pump and the difference in the absorption spectrum of the probe with and without the pump, ΔOD, is plotted. The TA spectra are corrected for the chirp in the probe pulse.

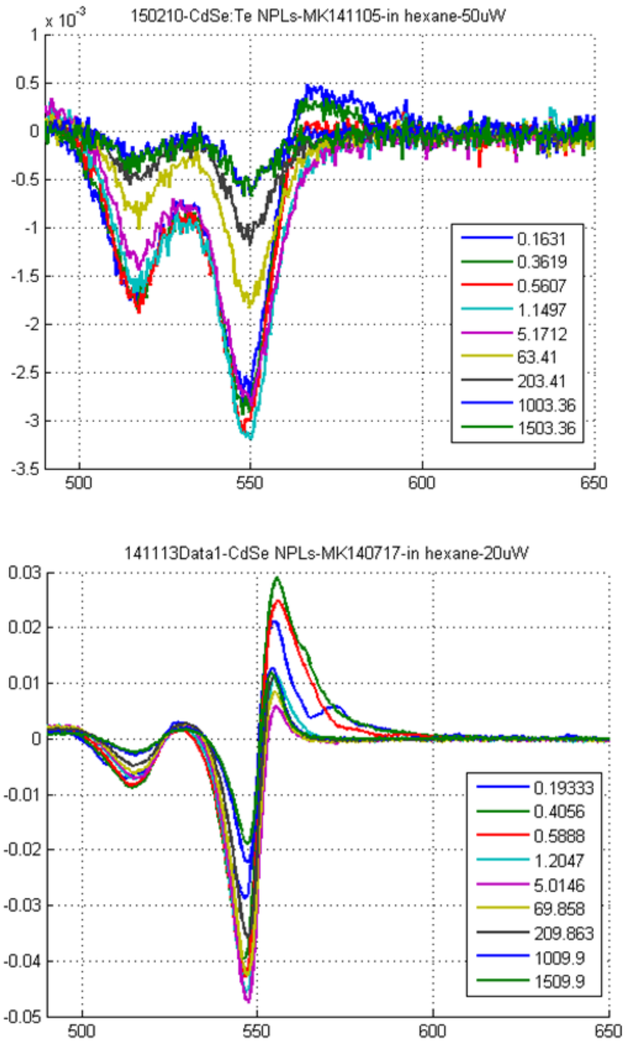

Figure S9: Transient absorption measurements at different time delays for the CdSe:Te NPLs (a) and for the undoped NPLs (b) measured in a hexane solution with a pump power of 50 uW (20uW for the undoped sample) corresponding to 0.5 absorbed photons per NPL. A bleach of the HH and LH peaks is seen for both the doped and undoped samples occurring due to state filling. The HH appears shifted by 12 meV in comparison to the undoped NPLs, identical to what is seen in the linear absorption spectrum. However, while the undoped sample shows only induced absorption in the range of 550nm to 575 nm assigned to charge transfer to surface defect states or

a formation of a charged trions, the doped sample shows a rapid transition from induced absorption to bleach between 550 nm to 600 nm within the first 1 ps after the pump. Follows the red tail seen in the linear absorption spectrum related to the Te states, indicating a fast cooling into long lived Te states.

Spectral Peak Asymmetry

In order to quantify the asymmetry in the PL spectra of single particles each curve was fit with a sum of three Gaussian function to reduce the effects of noise and background. The measure of asymmetry was then defined as:

$$asym = \frac{I_{left} - I_{right}}{I_{left} + I_{right}} \cdot 100\%$$

Where I_{left} is the integral over the spectrum from 0 to the peak wavelength and I_{right} is the same integral from the peak wavelength to infinity.

Extracting Lifetime Information from Single Particle Measurements

Single particle PL transients were generally not single exponential curves with at least three components required to fit the data well. In solution PL transient measurements the excitation power is much weaker and single NCs are not continuously excited. As a result blinking has very little effect on such measurements. It is therefore appropriate to compare solution PL transients to the ones obtained for single particles at the ‘On’ state. In our analysis each single particle trace is thresholded to extract periods in which the NPL was in the ‘On’ state and only those photon detections are used to construct the PL transient. The curves for lightly and heavily doped NPLs appearing in Figure 4 of the paper are the result of averaging over ‘On’ state single particle PL transients.

Photoluminescence Excitation (PLE) of doped NPLs

Photoluminescence Excitation (PLE) Measurements were carried out using a Hamamatsu Quantaurus-QY system. The emission was collected for 10nm detection windows centered at 570nm, 625nm and 670nm. The PLE measurements completely follow the features of the absorption spectrum whether detecting at the band edge emission wavelength (570nm) or at the red shoulder (~625nm).

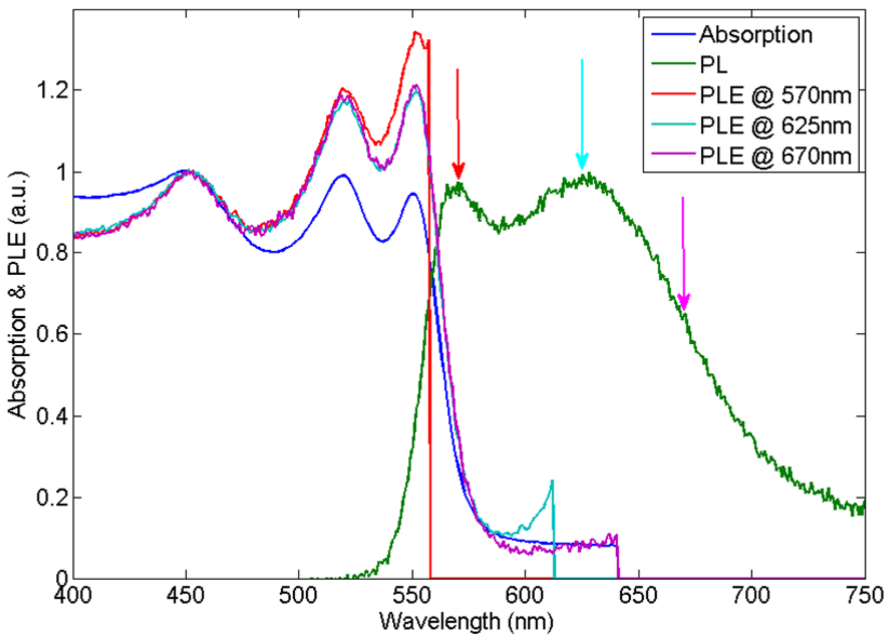

Figure S10: Photoluminescence Excitation (PLE) spectra for Te-TOP doped NPLs. Each arrow points to the PL wavelength used for PLE in the corresponding colored curve.
